# An intubated 7‐month‐old infant with a retropharyngeal abscess and multidrug‐resistant *Streptococcus mitis*

**DOI:** 10.1002/ccr3.2528

**Published:** 2019-11-06

**Authors:** Yoshihisa Watanabe, Yoshiro Nagao, Hisashi Endo, Ichiro Yamane, Masaaki Hirata, Kuniya Hatakeyama

**Affiliations:** ^1^ Department of Pediatrics Fukuoka Tokushukai Hospital Kasuga Japan

**Keywords:** age, antibiogram, antimicrobial resistance, antimicrobial susceptibility, deep neck infection, pediatrics, viridans streptococcus

## Abstract

The profile of antimicrobial resistance (ie, antibiogram) may be disparate between children and adults. An infant developed severe deep neck infection with a multidrug‐resistant microbe. The microbe was more drug‐resistant in children than in adults, in our hospital. Treatment of a child should be guided by the antibiogram obtained from children.

## Graphical abstract

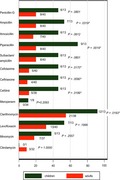

## 1 INTRODUCTION

A 7‐month‐old girl developed retropharyngeal abscesses and was intubated. Culture of the abscess yielded multidrug‐resistant *Streptococcus mitis.* The antibiogram in our hospital showed that *S mitis* in children was more resistant to antimicrobials than *S mitis* in adult patients. This case underlines the potential utility of age‐stratified hospital‐based antibiogram.

Retropharyngeal abscess is rare in pediatric population (eg, 4 per 100 000 children per annum in the United States).^1^ Retropharyngeal abscess is a life‐threatening situation because the abscess compresses the respiratory tract. The most common causative agent is viridans streptococci.^2^, ^3^, ^4^, ^5^ We report a 7‐month‐old patient with retropharyngeal abscesses caused by *Streptococci mitis*, a species of viridans streptococci. She was intubated, and the abscesses were surgically incised. Although the *S mitis* was resistant to many antibiotics, treatment with meropenem and clindamycin was successful.

## 2 CASE HISTORY/EXAMINATION

In 2016, a 7‐month‐old female infant without a remarkable medical history presented with a 39°C fever and wheezing. The patient had developed a low‐grade fever and cough 2 weeks prior. Subsequently, she developed a high fever and came to our hospital. She did not use antimicrobials in the recent past. On presentation, the patient's pharynx and tonsils were reddened, but the tonsils were not swollen. There was no palpable mass on her neck. Wheezing was heard in both lungs, and retracted breathing was noted. Although we did not notice any abnormalities in the chest X‐ray, laboratory tests were remarkable for a highly inflammatory profile: white blood cell count of 33 300/μL (79% neutrophils) and C‐reactive protein of 202 mg/L. Bacterial culture of the pharyngeal aspirate subsequently yielded *Haemophilus influenzae, Klebsiella oxytoca*, and *Streptococcus pneumoniae*. She was admitted (day 1) with a tentative diagnosis of severe acute pharyngitis, while an alternative differential diagnosis was deep neck infection. From day 1, we started intravenous cefotaxime at 150 mg/kg/day.

**Figure 1:**
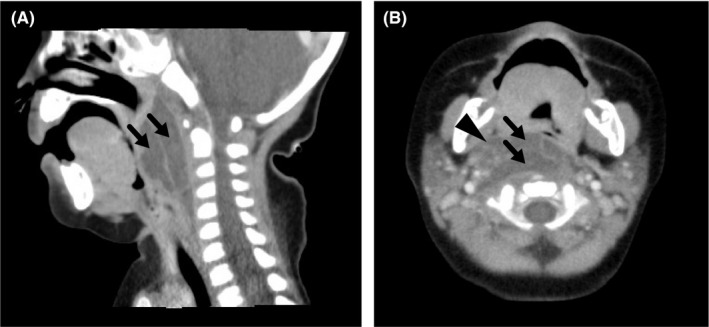
Retropharyngeal abscess and peritonsillar abscesses. The upper respiratory tract is completely compressed by retropharyngeal abscesses (arrows) and a peritonsillar abscess (triangle), as shown in sagittal (A) and coronal (B) sections

On day 2, however, her respiratory distress worsened, with severe retractions and respiratory acidosis. Enhanced computerized tomography (CT) revealed a right‐sided peritonsillar abscess and retropharyngeal abscesses (Figure 1). She was intubated immediately. An incision of the posterior pharyngeal wall yielded copious pus.

Empirically, we replaced cefotaxime with 10 mg/kg/day of meropenem from day 3. Additionally, on day 5, 300 mg/kg/day of ampicillin was started. A bacterial culture of the pus from the retropharyngeal abscess, which was incised on day 2, identified *Streptococcus mitis* on day 6. We replaced ampicillin with 40 mg/kg/day of clindamycin from day 8. Subsequently, a drug susceptibility test using an EIKEN FROZEN PLATE (Eiken Kagaku) reported resistance of the *S mitis* to multiple drugs (Table 1). The patient was extubated on day 10. Culture of the blood sample, which was collected on day 1, did not yield any microbe. She was discharged on day 24, with 9 mg/kg/day of oral tebipenem prescribed for 14 days. She has not had any relapse or severe infection to date.

**Table 1:** Susceptibility and resistance of the *Streptococcus mitis* identified in the retropharyngeal abscess of the present case to antibiotic drugs

Antibiotics	MIC^a^ defined as being susceptible (µg/mL)	MIC^a^ in the present patient (µg/mL)	Susceptibility^b^
1. Penicillins			
Penicillin‐G	≤0.12	2	I
Ampicillin	≤0.25	2	I
Amoxicillin	≤0.25	1	I
Piperacillin	≤0.25	1	I
Sulbactam/ampicillin	≤0.12/0.25	2	I
2. Cephalosporins			
Cefotaxime	≤1	>4	R
Ceftriaxone	≤1	>4	R
Cefditoren	≤0.5	>1	R
Cefdinir	≤0.5	>1	R
Cefcapene	≤0.5	>2	R
3. Carbapenems			
Meropenem	≤0.5	025	S
Panipenem	≤0.12	<0.12	S
4. Macrolides			
Azithromycin	≤0.5	>4	R
Clarithromycin	≤0.25	16	R
5. Quinolones			
Levofloxacin	≤2	>8	R
Tosufloxacin	≤0.5	>1	R
6. Tetracyclines			
Minomycin	≤2	<0.25	S
7. Lincosamides			
Clindamycin^c^			S

a Minimum inhibitory concentration (MIC).

b Resistant (R), intermediate (I), and susceptible (S).

c Susceptibility to clindamycin was estimated by Sensi‐Disk (BD; Franklin Lakes, NJ, USA) at the request of the physician.

## 3 DISCUSSION

Our patient initially showed only mild signs of an upper respiratory infection, but quickly deteriorated. Peritonsillar and retropharyngeal abscesses were diagnosed by enhanced CT. In our facility, 159 cases of peritonsillar abscess were treated between 2013 and 2017. During this period, only 11 cases of retropharyngeal abscess were treated here (Table 2). The rarity of this diagnosis may be due partly to the fact that it occurs in the deep tissue and relies upon imaging studies, particularly enhanced CT, to detect. Airway management was invasive: 5 cases (45%) were either intubated or tracheotomized. Treatment was surgical in most of the cases: 9 cases (82%) were incised.

**Table 2:** Profiles of 11 cases of retropharyngeal abscess who were diagnosed and treated in our hospital between 2013 and 2017

Age	Mean: 53 y; median: 63 y (range: 7 mo–81 y)
Mode of diagnosis^a^	Enhanced CT (8 cases), laryngoscopy (2), nonenhanced CT (1)
Airway management	Noninvasive (6), intubation (3), tracheotomy (2)
Treatment	Incision and drainage (9), supportive (2)
Antimicrobials^b^	Sulbactam/ampicillin (6), cefmetazole (4), clindamycin (4), cefotaxime (2), ampicillin (1), meropenem (1)
Number of intravenous antimicrobials^b^	Two (2), three (1), four (1), and one (7)
Bacteria in abscess	*Prevotella* (6), Viridans streptococcus (3), *Peptostreptococcus* (2), *Fusobacterium* (1)

a Computed tomography (CT).

b On average, a patient was treated with 1.6 antimicrobial drugs.

Our patient was also intubated and incised. *S mitis*, which was cultured from the abscess, was resistant to multiple antimicrobials. *S mitis* belongs to viridans streptococci species, which are commensal to the oral cavity.^6^, ^7^ Viridans streptococci are the most common cause of deep neck infections, followed by *Staphylococcus* species and anaerobes.^2^, ^3^, ^4^, ^5^ Viridans streptococci are assumed to be susceptible to penicillins and cephalosporins.^8^ Among viridans streptococci, however, *S* *mitis* is known to be highly resistant to multiple antibiotic drugs.^9^, ^10^, ^11^, ^12^, ^13^, ^14^, ^15^, ^16^, ^17^ Cephalosporin resistance is highly prevalent in *S mitis* in Japan.^18^ In addition, *S mitis* possesses various molecular strategies to compete with other bacteria in the oropharynx, promoting its virulence in opportunistic infections.^7^, ^19^ Consistently, infections with *S mitis* are associated with high morbidity and mortality, particularly among immunocompromised patients.^20^, ^21^, ^22^, ^23^, ^24^, ^25^ In contrast, our patient was immunocompetent and had not taken antimicrobials prior to this episode. This led us to review the medical records from our facility to understand the microbes that cause retropharyngeal abscess, especially *S mitis*.

Table 2 reveals that retropharyngeal abscesses frequently yielded anaerobes, including *Prevotella, Peptostreptococcus*, and *Fusobacterium*, in patients from our hospital. Viridans streptococci were identified in three patients, including the present case. Three patients were infected with more than one of these four microbial groups. In particular, one patient was infected with all four microbial groups. These microbes, other than the *S mitis* in the present case, were susceptible in vitro to cefmetazole or sulbactam/ampicillin (data not shown). As a result, treatment with these antibiotic drugs had been effective in all cases, except the case reported here.

In contrast, *S mitis* in our patient was highly resistant to cephalosporins, macrolides, and fluoroquinolones and was intermediately resistant to penicillins (Table 1). This resistance may explain why our initial treatment with cefotaxime was not effective. Instead, *S mitis* in the present case was susceptible to meropenem and clindamycin. Therefore, our empirical choice of these drugs was fortunately adequate.

**Figure 2:**
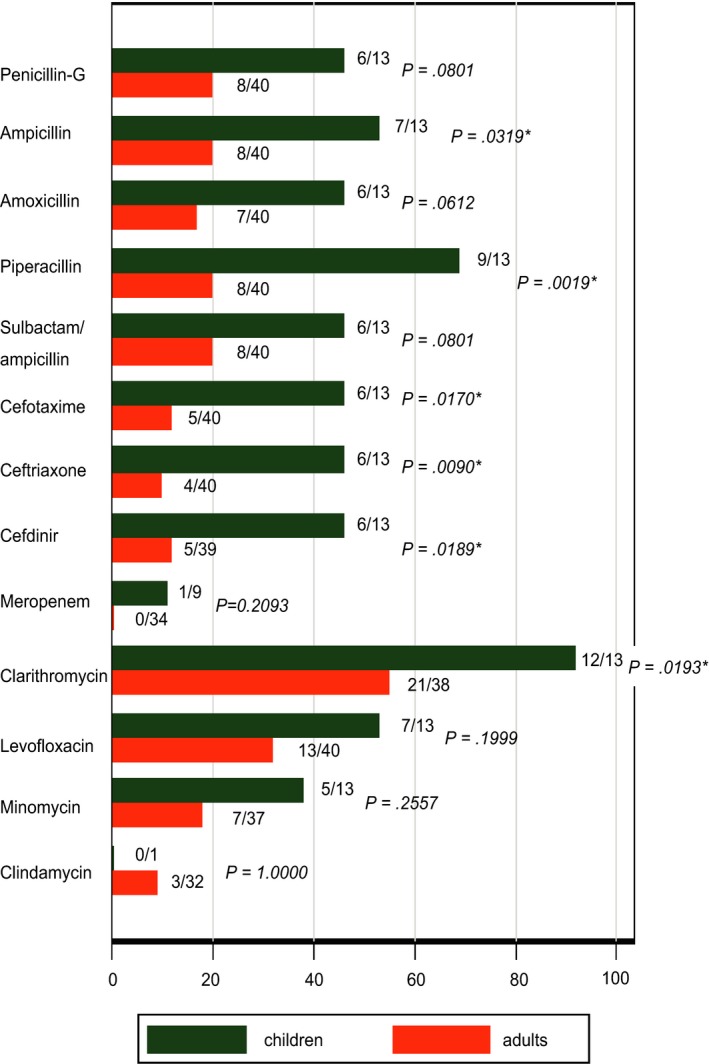
Antibiogram of *Streptococcus mitis* in our hospital, stratified by the age‐group. The percentage of resistant or intermediately resistant isolates, in the total number of isolates, is stratified by the age‐group: children (15 years or younger) or adult. To the right of each bar, the denominator and the numerator represent the total number of isolates and the number of resistant/intermediately resistant isolates, respectively. Statistically significant difference (ie, *P* < .05) in the percentage is indicated with an asterisk. *P*‐values were computed by two‐sided Fisher's exact test

The clinical microbiology laboratory of our hospital regularly issues the antibiogram—the profile of resistance and susceptibility to antimicrobials, based upon the bacterial culture database (La‐vietal MB^®^, Sysmex) which was introduced into our hospital in 2012. Upon our request, the clinical microbiology laboratory extracted records, accumulated between 2013 and 2017, that contained “*S mitis*”. We examined the case record from the electric medical record system, corresponding to these bacteriological records. As is shown in Table 3, there was no significant difference in the origin of samples between pediatric and adult patients (*P* = .4080 by two‐sided Fisher's exact test). The antibiogram for *S mitis*, stratified by age‐group (ie, pediatric patients of 15 years or younger, or adult patients), was compiled. This age‐stratified antibiogram showed that *S mitis* in our facility was more drug‐resistant in the children than in the adults (Figure 2). This may give a clue to our initial question: why retropharygeal abscess, a very rare disease in children, occurred in a healthy infant? The high prevalence of antimicrobial resistance of *S mitis* in the children in our community may have increased the probability that a healthy child encounters a multidrug‐resistant strain of this species which caused a severer disease than susceptible strains.^26^ It was previously suggested that frequent use of antibiotic treatment in pediatric and immno‐compromised populations have increased the rate of drug‐resistant viridans streptococci in these populations.^13^, ^27^

**Table 3:** Compositions of specimens with identified *Streptococcus mitis* from pediatric and adult patients between 2013 and 2017

Sample collection site^a^	Pediatric patients	Adult patients
Blood/spinal fluid	6 (35%)	19 (39%)^b^
Urine	3 (18%)	14 (29%)
Sputum/nasal swab	4 (24%)^c^	4 (8%)
Wound/pus/drainage	4 (24%)	12 (24%)
Total	17 (100%)	49 (100%)

a Three patients yielded *S mitis* from multiple body sites. As a result, 61 patients produced 66 isolates.

b One of these isolates was from the spinal fluid.

c One of these isolates was from nasal swab.

The utility of hospital‐based antibiogram in assisting in the selection of antimicrobial drug has been well recognized.^28^ In addition, it has been reported that the age greatly affects the profile of antimicrobial resistance.^29^, ^30^, ^31^ Despite the fact that the use of hospital‐based antibiogram is increasingly common, age‐stratified antibiogram was rarely utilized. Age‐specific information of antimicrobial resistance may provide useful input to the pediatric practice.

## 4 CONCLUSION

We reported a pediatric case of deep neck infection caused by *S mitis* that was resistant to multiple antimicrobials. Antibiogram stratified by age‐group suggested that *S mitis* was more drug‐resistant in children than in adults, in our hospital. Age‐specific and locale‐specific profile of antimicrobial resistance will be useful in guiding the treatment of children with severe infections.

## CONFLICTS OF INTEREST

The authors have no conflicts of interest to declare.

## AUTHOR CONTRIBUTIONS

YW: involved in the treatment of the patient and drafted the manuscript. YN: edited the manuscript. HE and IY: involved in the management of the patient. MH: endorsed the primary responsibility for the management of the patient. KH: conceptualized this report. All the authors have read the manuscript and approved its submission.

## INFORMED CONSENT

Written informed consent was obtained from her guardians.
